# Strenuous swimming raises blood non-enzymatic antioxidant capacity in rats

**DOI:** 10.1590/1414-431X2022e11891

**Published:** 2022-02-28

**Authors:** G. Godoy, P.B. Travassos, M.M. Antunes, C.C. Iwanaga, A.B. Sá-Nakanishi, R. Curi, J.F. Comar, R.B. Bazotte

**Affiliations:** 1 Programa de Pós-Graduação em Ciências Farmacêuticas, Universidade Estadual de Maringá, Maringá, PR, Brasil; 2 Programa de Pós-Graduação em Ciências Biológicas, Universidade Estadual de Maringá, Maringá, PR, Brasil; 3 Programa de Pós-Graduação Interdisciplinar em Ciências da Saúde, Universidade Cruzeiro do Sul, São Paulo, SP, Brasil; 4 Seção de Produção de Imunobiológicos, Centro Bioindustrial, Instituto Butantan, São Paulo, SP, Brasil

**Keywords:** Redox state, Physical exercise, Oxidative stress, Exhaustion, Reactive oxygen species, Lactate

## Abstract

The non-enzymatic antioxidant system protects blood components from oxidative damage and/or injury. Herein, plasma non-enzymatic antioxidant capacity after acute strenuous swimming exercise (Exe) and exercise until exhaustion (Exh) was measured in rats. The experiments were carried out in never exposed (Nex) and pre-exposed (Pex) groups. The Nex group did not undergo any previous training before the acute strenuous swimming test and the Pex group was submitted to daily swimming for 10 min in the first week and 15 min per day in the second week before testing. Plasma glucose, lactate, and pyruvate were measured and plasma total protein sulfhydryl groups (thiol), trolox equivalent antioxidant capacity (TEAC), ferric reducing ability of plasma (FRAP), and total radical-trapping antioxidant parameter (TRAP) levels were evaluated. There were marked increases in plasma lactate concentrations (Nex-Control 1.31±0.20 *vs* NexExe 4.16±0.39 *vs* NexExh 7.19±0.67) and in thiol (Nex-Control 271.9±5.6 *vs* NexExh 314.7±5.7), TEAC (Nex-Control 786.4±60.2 *vs* NexExh 1027.7±58.2), FRAP (Nex-Control 309.2±17.7 *vs* NexExh 413.4±24.3), and TRAP (Nex-Control 0.50±0.15 *vs* NexExh 2.6±0.32) levels after acute swimming and/or exhaustion. Also, there were increased plasma lactate concentrations (Pex-Control 1.39±0.15 *vs* PexExe 5.22±0.91 *vs* PexExh 10.07±0.49), thiol (Pex-Control 252.9±8.2 *vs* PexExh 284.6±6.7), FRAP (Pex-Control 296.5±15.4 *vs* PexExh 445.7±45.6), and TRAP (Pex-Control 1.8±0.1 *vs* PexExh 4.6±0.2) levels after acute swimming and/or exhaustion. Lactate showed the highest percent of elevation in the Nex and Pex groups. In conclusion, plasma lactate may contribute to plasma antioxidant defenses, and the TRAP assay is the most sensitive assay for assessing plasma non-antioxidant capacity after strenuous exercise.

## Introduction

Cells have evolved a sophisticated enzymatic antioxidant system that includes enzymes such as glutathione peroxidase, glutathione reductase, superoxide dismutase, and catalase that scavenge and prevent reactive oxygen species (ROS) and reactive nitrogen species (RNS) accumulation (1). Interestingly, plasma has been reported to have lower antioxidant enzyme activities than the intracellular environment (2).

This observation is due to the fact that plasma is continuously exposed to ROS (3) and contains non-enzymatic antioxidant substances, such as albumin, ascorbic acid, α-tocopherol, bilirubin, creatinine, and uric acid. This non-enzymatic antioxidant system protects blood components from oxidative damage and/or injury (4-7). Several studies have suggested that this increased antioxidant capacity (8-811) is needed to prevent oxidative stress (12).

Intense and exhaustive exercise increases oxygen consumption and demand and stimulates oxidative phosphorylation and ROS generation (13,14), thereby inducing oxidative stress. Excessive ROS and RNS production can modify and damage DNA, lipids, and proteins (1), negatively influencing various physiological functions and processes (4-7). Indeed, oxidant-induced (i.e., oxidative) damage has been linked to the onset and progression of diabetes, cardiovascular diseases, arthritis, cancer, and other diseases and disorders (4-6).

The redox state, especially antioxidant defenses, in exercising experimental animals and humans has been extensively studied (3,8,11,13). However, these studies employed various exercise intensities, durations, and training schedules, with participants of both genders and different ages (8-11,13-15). Consequently, this heterogeneity limits and jeopardizes the evaluation of the role of antioxidant defenses during strenuous exercise.

The present study assessed plasma lactate levels after exercise and exhaustion and their potential association with antioxidant defenses. Furthermore, the plasma non-enzymatic antioxidant capacity was evaluated in rats after acute strenuous swimming and swimming until exhaustion using four different methods: total protein sulfhydryl groups (thiol), trolox equivalent antioxidant capacity (TEAC), ferric reducing ability of plasma (FRAP), and total radical-trapping antioxidant parameter (TRAP).

## Material and Methods

### Ethical approval

The experimental procedures followed the international laws and the institutional guidelines for practical animal care and were approved by the Ethics Committee of the State University of Maringá (CEUA protocol number 3659240315).

### Chemicals

The 2,2′-azo-bis-(2-amidinopropane) (ABAP), 2,2′-azino-bis-3-ethylbenzothiazoline-6-sulfonic acid (ABTS), dimethylsulfoxide (DMSO), 5,5′-dithiobis-2-nitrobenzenic acid (DNTB), 2,4-dinitrophenylhydrazine (DNPH), tripyridyltriazine (TPTZ), and 6-hydroxy-2,5,7,8-tetrametylchloraman-2-carboxylic acid (Trolox) were purchased from Sigma-Aldrich (USA).

### Animals

Male Wistar rats weighing from 210-240 g (7 weeks old) were obtained from the State University of Maringá breeding center and housed in a room with controlled temperature (22±1°C) and humidity (60±10%) and a 12-h light/dark cycle. The animals had free to access to standard rodent chow (Nuvital Nutrients S/A, Brazil) and water.

### Experimental protocol

**Figure 1:**
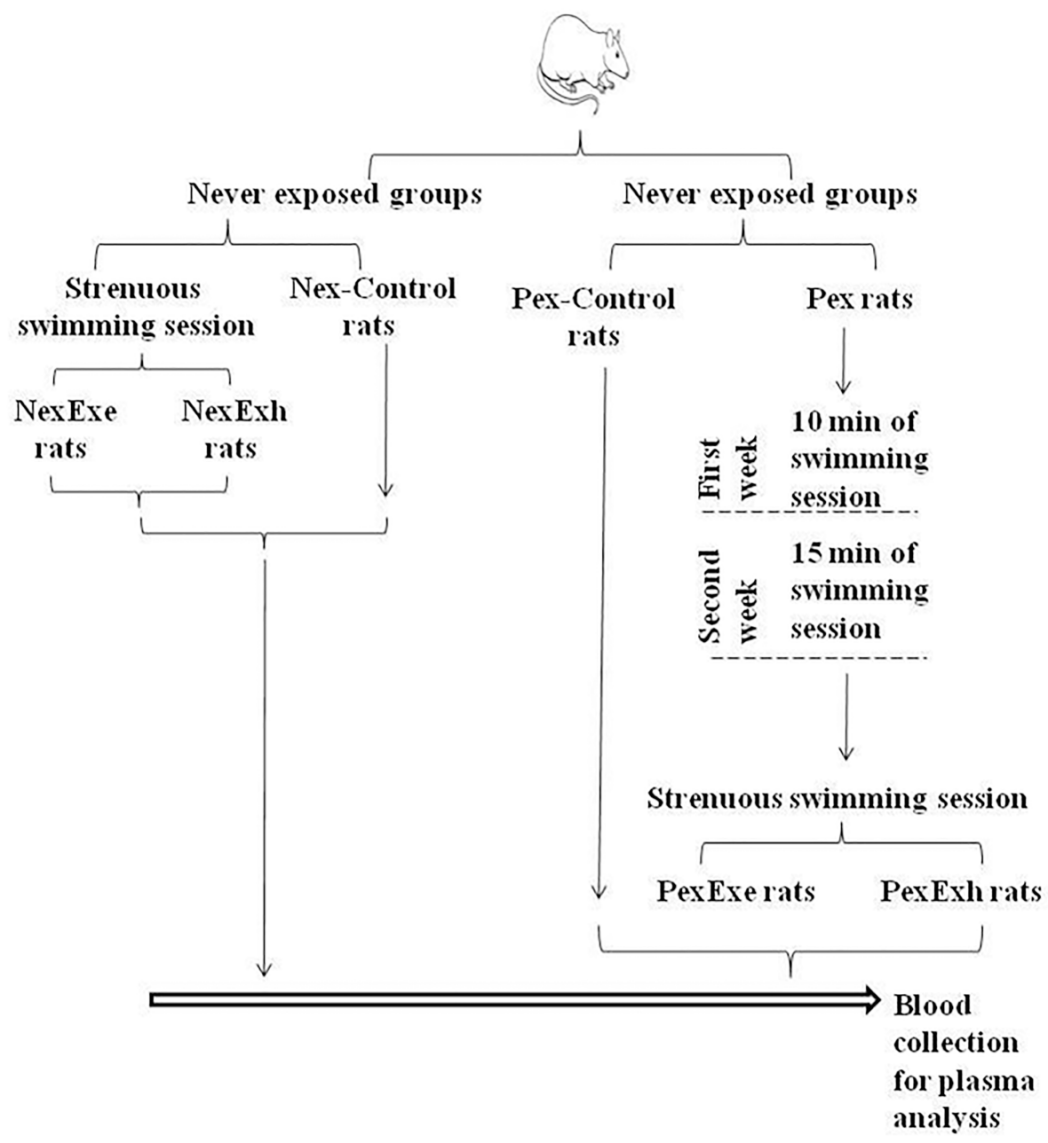
Experimental design. Left side: Never exposed (Nex) protocol. Two Nex rats were placed in the water tank, and once the first rat reached exhaustion (NexExh), the second rat was immediately removed from the water (NexExe). The control group (Nex-Control) consisted of rats placed in the water tank and immediately removed. Right side: Pre-exposed (Pex) protocol. In the first week, rats were submitted to 10-min daily exercise and in the second week, they swam for 15 min each day. On the last day, a pair of rats were left in the water for 15 min to swim (PexExe) or until exhaustion (PexExh). Control rats (Pex-Control) were placed in the water tank and immediately removed.

The experimental protocol was approved by an Institutional Review Board following the National Council for the Control of Animal Experimentation and is presented in Figure 1.

The swimming session was performed in cylindrical tanks (60×30 cm, 30 L capacity) with a water temperature of 31±1°C. Before each session, a load corresponding to 6% of the body weight was tied to the tail of the animals (15).

The never exposed group (Nex) was fasted overnight (15 h). Then, two rats were simultaneously placed in the swimming tank side by side for the acute swimming-test protocol. When the first rat was exhausted (NexExh), the second rat was also removed from the water (NexExe). Exhaustion was defined as failure to stay above the water surface, loss of symmetrical movements, and/or remaining underwater for more than 5 s (15). The control group (Nex-Control) consisted of overnight-fasted rats that were placed in the swimming tank and immediately removed.

Rats in the pre-exposed (Pex) group were submitted to 10-min daily swimming sessions for one week and 15-min daily swimming sessions for an additional week. Two days of rest were given between the first and second weeks of the pre-exposure protocol. On the final day of the second week, the Pex group was fasted overnight. Then a pair of rats were placed in the tank to swim for 15 min (PexExe) or until exhaustion (PexExh). A control group (Pex-Control) was fasted overnight, placed in the swimming tank, and immediately removed.

Following the swimming tests, all rats (Nex and Pex groups) were removed from the water, and their necks were dried to prevent hemolysis. Then, each animal was immediately euthanized by decapitation for blood collection. The time between removing the animals from the water and decapitation was less than 15 s. The swimming sessions for the Nex and Pex groups were performed on different days.

### Blood sample and biochemical analysis

After euthanasia, blood was collected in EDTA-containing tubes and immediately centrifuged at 1,700 *g* for 10 min at 4°C. Plasma glucose, lactate, and pyruvate concentrations were measured as previously described (16).

### Determination of plasma antioxidant parameters

The plasma's antioxidant capacity was assessed using four different methods: thiol content, TEAC, FRAP, and TRAP.

Thiol content was measured by spectrophotometry using DNTB as previously described (7). Briefly, an aliquot of plasma was incubated with a 90 mM TRIS buffer (pH 8.6), 5 mM EDTA solution. The initial absorbance was taken at 412 nm. Then, 10 mM DTNB was added, the samples were incubated in the dark for 15 min, and the absorbance was measured again. The thiol content was calculated using the molar extinction coefficient (ε) of 1.36×10^4^ M^-1^·cm^-1^. The results are reported as nmol/mL of plasma (17).

The spectrophotometric TEAC assay is based on hydrogen peroxide reacting with ABTS to form an ABTS cation (ABTS^+^), which strongly absorbs light at 734 nm. The plasma's ability to neutralize ABTS radicals causes a reduction in absorbance. The total antioxidant capacity was calculated from the standard curve prepared with Trolox, a water-soluble analog of vitamin E. The results are reported as nmol/mL of Trolox (7,18).

The FRAP method monitors the plasma-induced conversion of Fe^3+^ to Fe^2+^. The plasma's reducing capacity was measured spectrophotometrically at 595 nm using TPTZ and ferric chloride solutions. FRAP was calculated from a standard curve prepared with Trolox. The results are reported as nmol/mL of Trolox (19).

Lastly, using ABAP and luminol, the TRAP method detects the hydrosoluble and/or liposoluble antioxidants in the plasma by chemiluminescence (20). Herein, 20 µM and 200 µM ABAP were used as the free radical source to react with luminol and generate chemiluminescence. The reaction is inhibited by superoxide dismutase, catalase, and vitamin E analogs. The addition of 70 µL of plasma attenuated the chemiluminescence to basal levels for a period proportional to the plasma levels of TRAP until the luminol radicals are regenerated. The system was calibrated with Trolox. By comparing the induction time after the addition of known concentrations of Trolox and plasma, it is possible to obtain TRAP values as equivalent Trolox levels. The peak chemiluminescence emission was detected with a Glomax luminometer (Turner Designs TD 20/20, USA), and TRAP values were calculated as previously described (21). The results are reported as nmol/mL of Trolox.

### Statistical analysis

All statistical analyses were performed with the Graph-Pad Prism Software version 5.0 (GraphPad Software, USA). The data are reported as means±SE. The results were analyzed using one-way ANOVA with the Tukey test for *post hoc* comparisons. The level of significance was set at a P-value of <0.05.

## Results

### Plasma glucose, lactate, and pyruvate levels in the never exposed group

**Figure 2:**
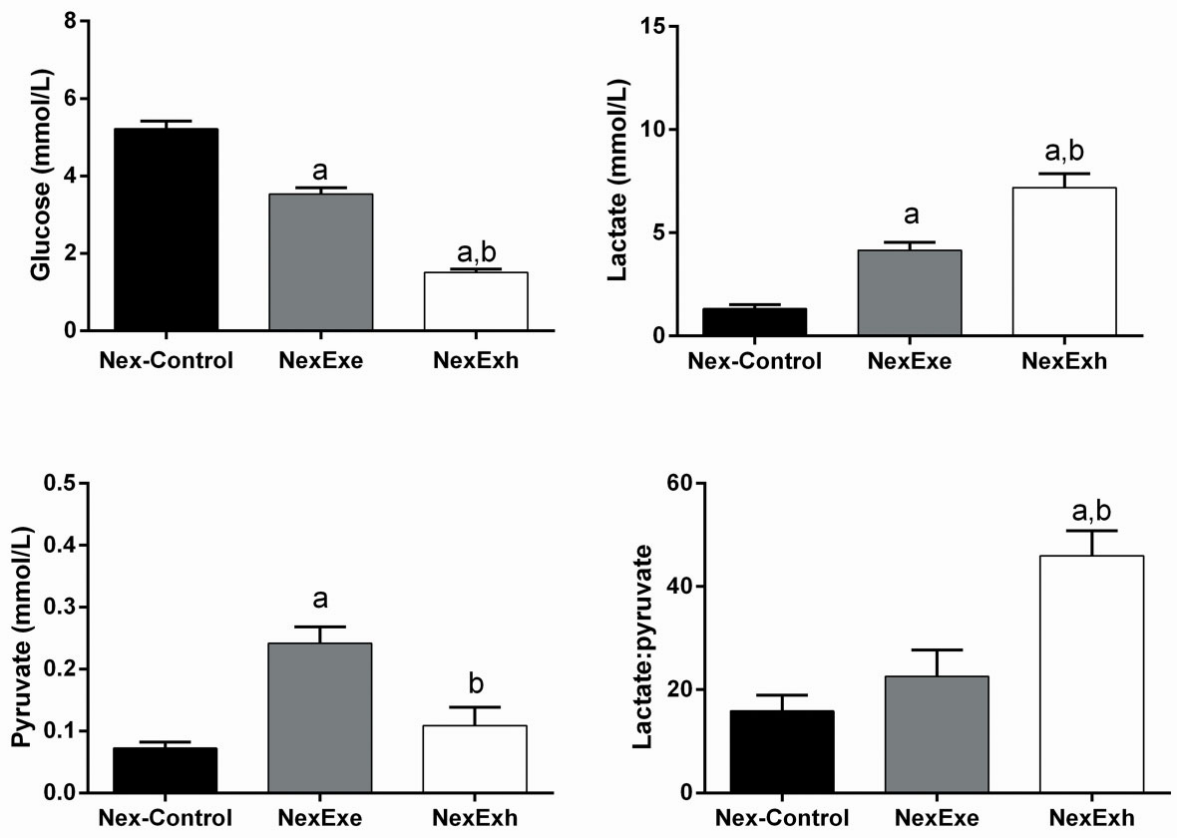
Plasma metabolites measurements in the never exposed (Nex) group. Effect of an acute strenuous swimming session on plasma glucose, lactate, pyruvate concentrations, and the lactate:pyruvate ratio in overnight fasted rats. Two rats were placed in the water tank. Once the first rat reached exhaustion (NexExh), the second rat was immediately removed from water (NexExe). The control rats (Nex-Control) were placed in the water tank and immediately removed. Data are reported as means±SE (n=7 animals per group). ^a^P<0.05 compared with the Nex-Control group; ^b^P<0.05 compared with the NexExe group (one-way ANOVA with the Tukey test for *post hoc* comparisons).

The NexExe and NexExh rats exhibited lower glucose levels than the Nex-Control rats (P<0.05). Moreover, the glucose levels of the NexExh rats were also reduced compared with NexExe rats (P<0.05). The plasma lactate levels of the NexExe and NexExh rats were 216.5% and 447.4% higher than the Nex-Control group (P<0.05) and significantly higher in NexExh rats compared with NexExe rats (P<0.05). The NexExe rats displayed elevated pyruvate levels compared with the Nex-Control and NexExh groups (P<0.05). While the pyruvate concentration in the NexExh group tended to be higher than in Nex-Control rats, this result failed to reach a level of statistical significance. The lactate:pyruvate ratio of the NexExh rats was elevated compared with Nex-Control and NexExe rats (P<0.05). On the other hand, the lactate:pyruvate ratios of the Nex-Control and NexExe groups were similar. (Figure 2).

### Plasma glucose, lactate, and pyruvate levels in the pre-exposed group

**Figure 3:**
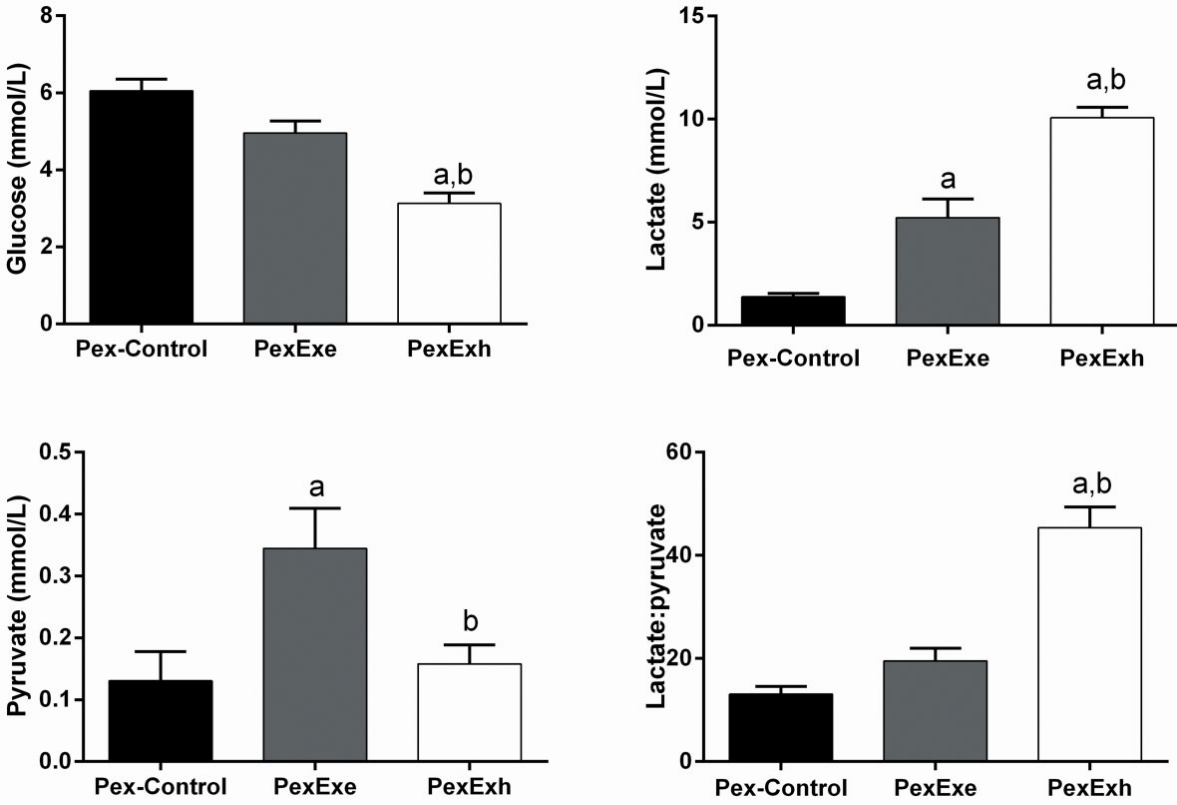
Plasma metabolite measurements in the pre-exposed (Pex) group. Effect of an acute strenuous swimming session on plasma glucose, lactate, pyruvate concentrations, and the lactate:pyruvate ratio in overnight fasted rats. Two rats were placed in the water tank on the last day of the pre-exposed protocol. The first rat swam for 15 min (PexExe) and the second rat swam until exhaustion (PexExh). The control rats (Pex-Control) were placed in the water tank and immediately removed. Data reported as means±SE (n=5-6 animals per group). ^a^P<0.05 compared with the Pex-Control; ^b^P<0.05 compared with the PexEx group (one-way ANOVA with the Tukey test for *post hoc* comparisons).

The PexExh rats had lower plasma glucose levels than the PexExe and Pex-Control rats (P<0.05), and the PexExe and Pex-Control rats presented similar levels. The plasma lactate levels in the PexExe and PexExh groups were 219.1% and 470.5% higher than the Pex-Control rats, respectively (P<0.05) and significantly higher in the PexExh rats compared with the PexExe group (P<0.05). The pyruvate levels were higher in the PexExe rats compared with Pex-Control and PexExh rats (P<0.05), which were statistically equivalent. Furthermore, the lactate:pyruvate ratio of the PexExh rats was higher (P<0.05) than the Pex-Control and PexExe rats. The lactate:pyruvate ratio in the PexExe group was not significantly different from that in Pex-Control animals. (Figure 3).

### Plasma non-enzymatic antioxidant capacity in the never exposed group

The antioxidant capacities measured by the thiol assay were 6.3% and 15.7% higher in the plasma of NexExe and NexExh rats, respectively, compared with the Nex-Control rats. However, only the NexExh group was found to be significantly increased (P<0.05). Furthermore, the TEAC assay's antioxidant capacity was 17.6% and 30.7% higher in the plasma of the NexExe and NexExh rats than the Nex-Control rats. Similar to the thiol assay, only the NexExh group displayed significantly increased antioxidant capacity. The FRAP assay detected 4.2% and 33.7% increases in the antioxidant capacity of the plasma from NexExe and NexExh rats, respectively, compared with the Nex-Control rats. Lastly, the antioxidant capacities measured by the TRAP assay were 100% and 430% higher in the plasma of the NexExe and NexExh rats than in the Nex-Control rats (P<0.05).

**Figure 4:**
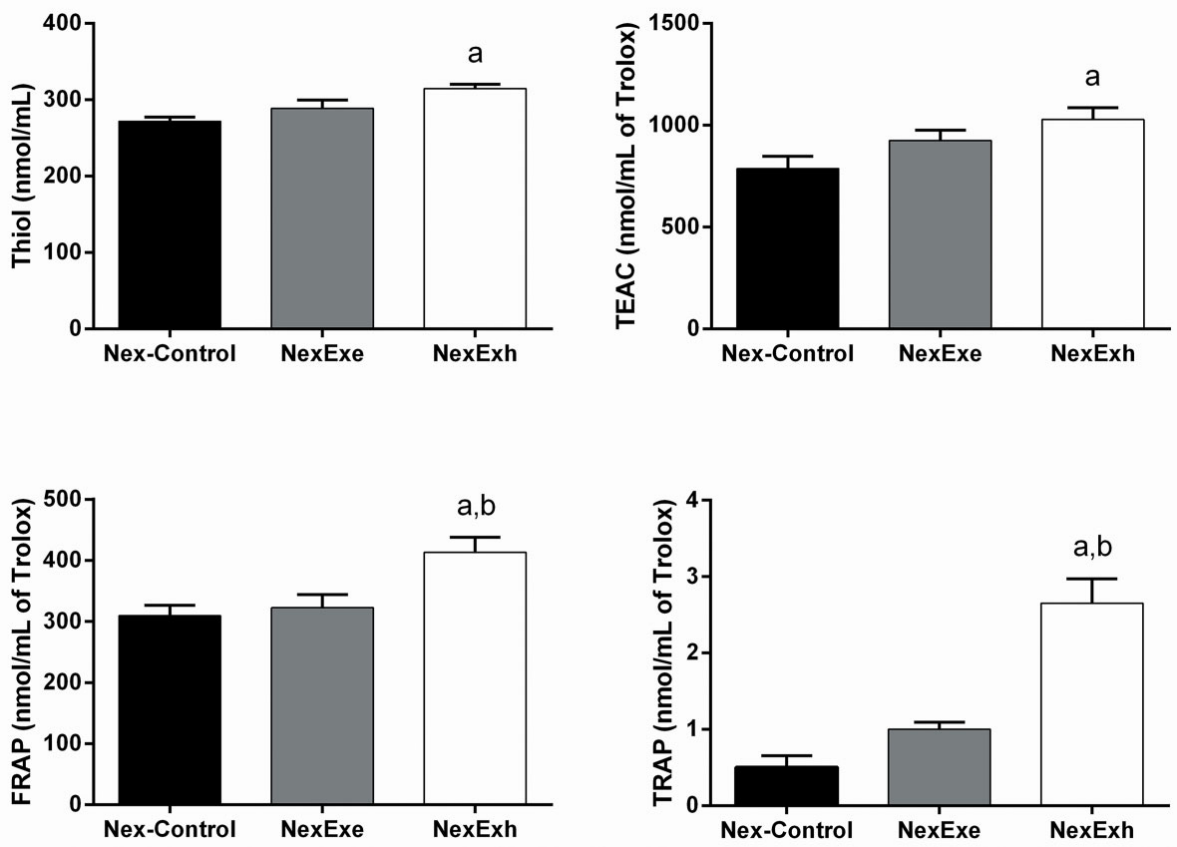
Plasma antioxidant capacity measurements in the never exposed (Nex) group. Effect of an acute strenuous swimming session on the plasma protein sulfhydryl group (thiol) content, Trolox equivalents antioxidant capacity (TEAC), ferric reducing ability of plasma (FRAP), and total radical-trapping antioxidant parameter (TRAP) in overnight fasted rats. Two Nex rats were placed in the water tank. Once the first rat reached exhaustion (NexExh), the second rat was immediately removed from water (NexExe). The control rats (Nex-Control) were placed in the water tank and immediately removed. Data reported as means±SE (n=7 animals per group). ^a^P<0.05 compared with the Nex-Control group; ^b^P<0.05 compared with the NexExe group (one-way ANOVA with the Tukey test for *post hoc* comparisons).

Overall, the thiol, TEAC, FRAP, and TRAP data indicated that the antioxidant capacity of the NexExh group was significantly greater than the Nex-Control group (P<0.05). Additionally, the FRAP and TRAP assays detected enhanced antioxidant capacity in NexExh rats compared with NexExe rats (P<0.05). (Figure 4).

### Plasma antioxidant capacity in the pre-exposed group

**Figure 5:**
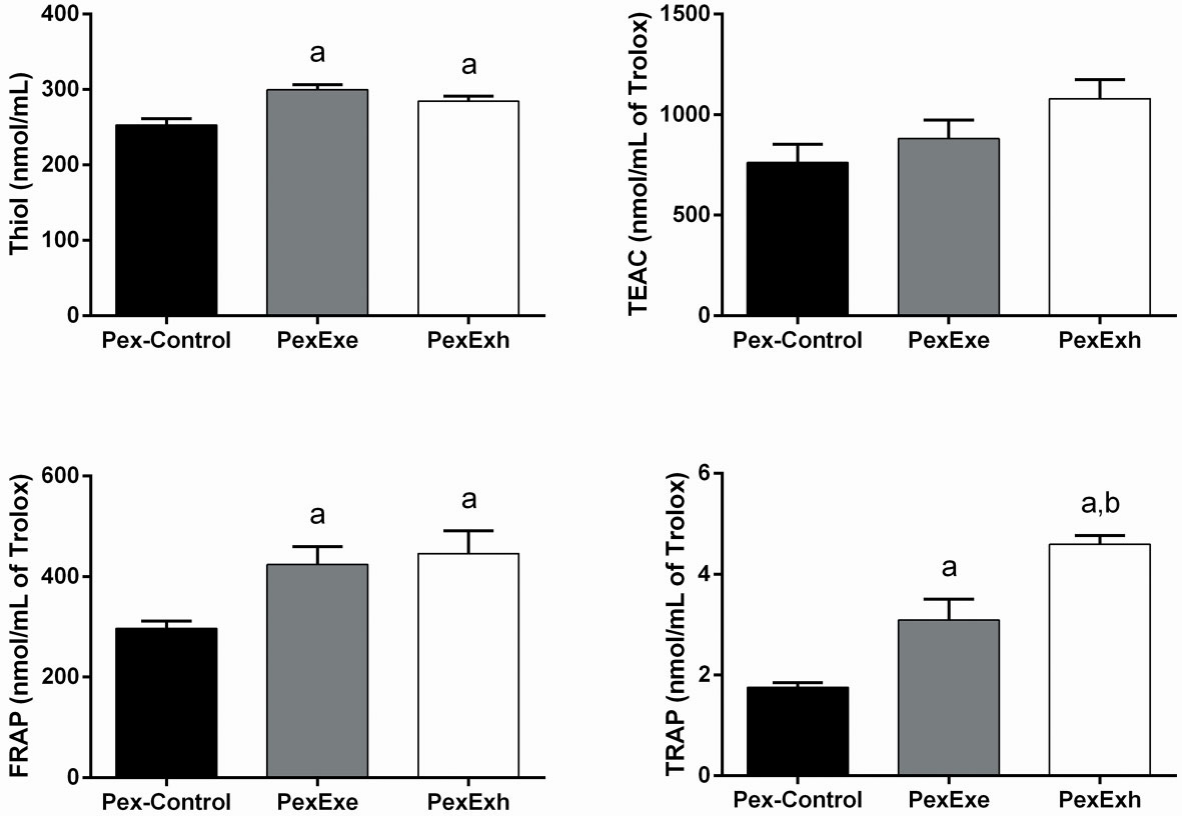
Plasma antioxidant capacity measurements in the pre-exposed (Pex) group. Effect of a strenuous swimming session on plasma protein sulfhydryl groups (thiol) content, Trolox equivalents antioxidant capacity (TEAC), ferric reducing ability of plasma (FRAP), and total radical-trapping antioxidant parameter (TRAP) in overnight fasted rats. The Pex group trained for two weeks. Two rats were placed in the water tank on the last day of the pre-exposed protocol. The first rat swam for 15 min (PexExe) and the second rat swam until exhaustion (PexExh). The control rats (Pex-Control) were placed in the water tank and immediately removed. Data are reported as means±SE (n=5-6 animals per group). ^a^P<0.05 compared with the Pex-Control group; ^b^P<0.05 compared with the PexExe group (one-way ANOVA with the Tukey test for *post hoc* comparisons).

In the Pex group, the thiol assay indicated that the antioxidant capacities of the PexExe and PexExh rats were increased by 18% and 13%, respectively, compared with the Pex-Control rats (P<0.05). The plasma TEAC levels were 15.5% and 41.5% higher for the PexExe and PexExh groups compared with Pex-Control rats; however, these differences failed to reach a level of statistical significance. The FRAP levels of PexExe and PexExh rats were 42% and 50% higher than the Pex-Control rats (P<0.05). (Figure 5).

The TRAP assay detected antioxidant capacity increases of 76% and 162% in the PexExe and PexExh groups compared with Pex-Control rats (P<0.05). Moreover, the TRAP levels of the PexExh group were significantly greater than that of the PexExe group (Figure 5).

## Discussion

**Figure 6:**
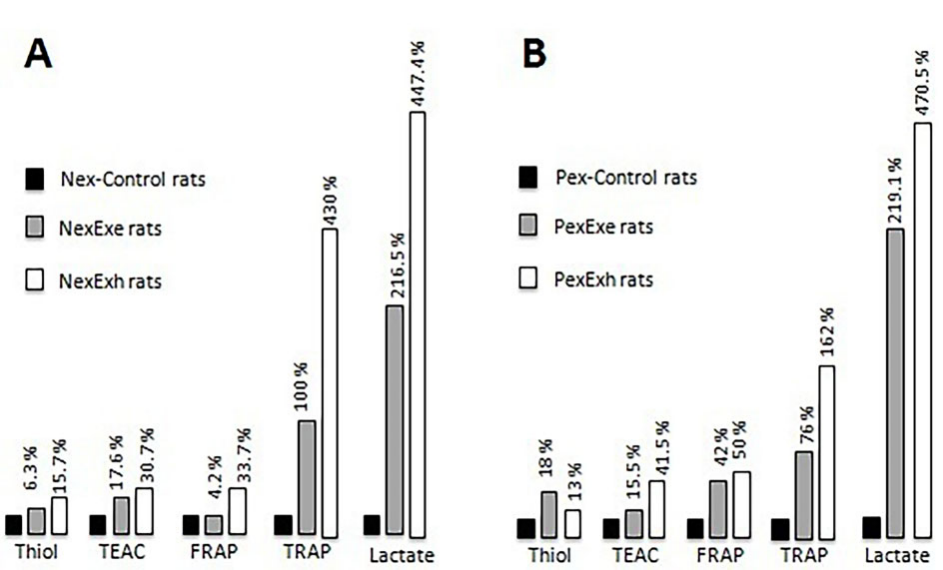
Summary of the antioxidant capacity experiments. **A**, Never exposed (Nex) group. **B**, Pre-exposed (Pex) group. Percent increase in thiol, TEAC, FRAP, TRAP, and lactate levels after exercise (Exe rats: gray bars) and exhaustion (Exh rats: white bars) compared with control groups (black bars). The increase in plasma lactate (Control rats *vs* Exe or Exh rats) was more pronounced than thiol, TEAC, FRAP, and TRAP levels in the Nex or Pex groups, thus, suggesting a potential role for lactate as an antioxidant after strenuous exercise and exhaustion. Thiol: protein sulfhydryl group content; TEAC: trolox equivalents antioxidant capacity; FRAP: ferric reducing ability of plasma; TRAP: total radical-trapping antioxidant parameter.

This study compared the rat plasma antioxidant capacity after performing an acute strenuous swimming test. A summary of the thiol, TEAC, FRAP, and TRAP assay results is presented in Figure 6. The histograms demonstrate that the TRAP and lactate levels were elevated in the Nex and Pex exercise and exhaustion groups compared with the Nex-Control and Pex-Control groups, respectively. It is worth mentioning that since the acute swimming-test protocols for the Nex and Pex groups were performed on different days, it was impossible to compare them. However, despite this limitation, there is a general tendency for similar plasma TRAP and lactate increases after acute swimming and exhaustion.

The plasma thiol levels represent the action of antioxidant molecules such as albumin and other proteins of low molecular weight (7,17). As expected, the plasma thiol levels in the Nex and Pex groups increased after exercise and exhaustion compared with the levels of the respective control group. However, the plasma thiol level increases were negligible compared with the plasma TRAP levels in both groups of animals.

It was previously reported that the plasma TEAC assay is suitable for measuring plasma non-enzymatic antioxidant defenses since TEAC is mainly composed of albumin and uric acid (22,23). In general, plasma TEAC levels were similar in Nex and Pex groups following exercise and exhaustion compared with the control groups. Similar to the thiol content results, the plasma TEAC increase was small compared with the increased plasma TRAP levels.

The plasma FRAP assay measures all antioxidant mechanisms that reduce the metal complex Fe^3+^-TPTZ ion (19). The plasma FRAP levels were higher in NexExe rats than in Nex-Control rats. Additionally, the plasma FRAP levels were higher in PexExe and PexExh rats than in Pex-Control rats. Like the plasma thiol and TEAC levels, the degree of elevation of plasma FRAP levels was small compared with plasma TRAP levels.

It was previously reported that the plasma TRAP assay could measure antioxidant mechanisms (24). Our results using Nex and Pex rats after exercise and exhaustion demonstrate that the plasma TRAP assay is the most robust method for assessing non-enzymatic antioxidant capacity, at least when compared with the thiol, FRAP, and TEAC assays. Additionally, this assay provides a better representation of the global antioxidant potential, assessing the synergistic contributions of urate, plasma proteins, ascorbate, and tocopherol to plasma's non-enzymatic antioxidant capacity (24). A previous study using the TRAP assay reported that the relative contributions of urate, plasma proteins, ascorbate, and tocopherol were between 35-65%, 10-50%, 0-24%, and 5-10%, respectively (25).

Additionally, our results demonstrates that a strenuous swimming session increased plasma lactate concentrations and the lactate:pyruvate ratio, an observation that was consistent with previous studies (15,26). The increased lactate:pyruvate ratio is indicative of changes in the redox state (i.e., NADH:NAD^+^ ratio) after exercise and exhaustion in the Nex and Pex groups. These changes are in response to increased muscle glycolysis and pyruvate-to-lactate conversion via lactate dehydrogenase (27). To our knowledge, this is the first study to identify a relationship between elevated plasma TRAP levels and lactate concentrations after intense exercise and exhaustion. In this sense, investigating the contribution of plasma lactate to the antioxidant defenses could provide valuable insights into the underlying mechanisms.

For many years, lactate was considered a metabolic residue (28,29). It is currently recognized for its role as a liver glucose precursor (15), an angiogenesis signal (28), an oxidative substrate in neurons (30), a memory consolidation molecule (31), and an antioxidant in human cells (32-34). Today, the well-established role of lactate as an antioxidant justifies its use as a food preservative (35). Despite the accumulating evidence, the role lactate plays in the plasma non-enzymatic antioxidant defenses remains unclear.

Similar to the results of our study, previous studies have shown that serum lactate levels increase proportionally with antioxidant capacity during short periods of high-intensity exercise (8,33). Additionally, there are several studies demonstrating lactate's antioxidant activity. For example, Anbar and Neta (36) first showed that lactate works as a hydroxyl radical (OH) scavenger. Groussard et al. (37) also reported that lactate could scavenge OH in addition to superoxide anions (O_2_^-^). The concentration-dependent antioxidant effect of lactate and its substantial increase during intense exercise and exhaustion reinforce the possibility that it functions as an antioxidant. Lactate-mediated OH scavenging was later expanded to pyruvate by Herz et al. (38). According to the authors, lactate-mediated OH reduction generates pyruvate, which scavenges O_2_^-^, H_2_O_2_, and OH through its conversion into acetate and CO_2_ (39,40). Therefore, lactate's role as an antioxidant must be considered since plasma lactate concentrations increase after exercise and exhaustion.

In conclusion, our study revealed that the plasma TRAP assay was more sensitive at assessing plasma antioxidant capacity following acute strenuous exercise and exhaustion than the thiol, TEAC, and FRAP assays. It is plausible that lactate substantially contributed to the non-enzymatic antioxidant defense system.
